# Dorsal approach in laparoscopic extended left hemi-hepatectomy: A case series

**DOI:** 10.1097/MD.0000000000037336

**Published:** 2024-03-01

**Authors:** Katsunori Sakamoto, Kohei Ogawa, Kei Tamura, Masahiko Honjo, Takahiro Hikida, Chihiro Ito, Miku Iwata, Akimasa Sakamoto, Mikiya Shine, Yusuke Nishi, Mio Uraoka, Tomoyuki Nagaoka, Naotake Funamizu, Yasutsugu Takada

**Affiliations:** a Department of Hepato-Biliary-Pancreatic and Breast Surgery, Ehime University Graduate School of Medicine, Shitsukawa, Toon, Ehime, Japan.

**Keywords:** extended left hemi-hepatectomy, Glissonean pedicle, laparoscopy, middle hepatic vein

## Abstract

**Rationale:**

The utility of the dorsal approach has been reported for laparoscopic left hemi-hepatectomy.

**Patient concerns:**

The aim of the present study is to show the usefulness of the dorsal approach for laparoscopic extended left-hemi-hepatectomy while ensuring safe identification of hepatic veins and dissection of the dorsal tumor margin.

**Diagnoses:**

Tumors requiring extended left hemi-hepatectomy.

**Interventions:**

After mobilization of the lateral sector and division of the Arantius plate, parenchyma above the Arantius plate is removed to expose the root of the middle hepatic vein and left hepatic vein. Each of these veins can be isolated separately either intra- or extra-hepatically. After removing the parenchyma on the cranial side of the left Glissonean pedicle continuous with the exposed hepatic veins, the left Glissonean pedicle is isolated using the Glissonean pedicle transection method. After division of the left hepatic vein and Glissonean pedicle, segment 4 (in which the main part of the tumor is commonly located) is dissected from the anterior plane of the paracaval portion of the caudate lobe by the dorsal approach, along with the hepatic hilum. Following dissection of the dorsal side of the tumor, and division of parenchyma from the anterior edge of the liver, the anterior Glissonean branches and middle hepatic vein are divided safely and the specimen is resected.

**Outcomes:**

Three patients underwent laparoscopic extended left hemi-hepatectomy, with no open conversions. Operative time and blood loss were 331 (concomitant with another partial hepatectomy), 277, and 315 minutes; and 200, 100, and 100 g, respectively. The postoperative courses were uneventful.

**Lessons:**

The dorsal approach maximizes the advantages of laparoscopic extended left hemi-hepatectomy and can be performed safely.

## 1. Introduction

Laparoscopic liver resection is now a widely accepted procedure with favorable outcomes.^[1,2]^ Laparoscopic anatomical liver resection is also becoming widespread, despite requiring difficult techniques.^[3,4]^ In advanced laparoscopic anatomical liver resection, left hemi-hepatectomy is suitable for the introduction of advanced laparoscopic hepatectomy due to its simplicity.^[5,6]^ The utility of the dorsal approach has been reported for laparoscopic left hemi-hepatectomy^[7,8]^; however, this procedure is considered difficult for tumors located in the postero-superior area of segment 4 because the root of the middle hepatic vein runs close to the tumor.^[9]^ Similarly, laparoscopic extended left hemi-hepatectomy is a difficult procedure because a more advanced technique is often necessary to resect the tumor with oncological safety. Laparoscopic extended left hemi-hepatectomy often requires procedures such as parenchymal dissection along the hilar plate, appropriate dissection of anterior Glissonean branches, and safe division of the middle hepatic vein to enable removal of the tumor with a negative surgical margin. The dorsal approach may provide additional advantages in such cases. The aim of this study is to show the usefulness of the dorsal approach during laparoscopic extended left-hemi-hepatectomy.

## 2. Methods

### 2.1. Operative procedure (Supplemental video)

**Figure 1.**
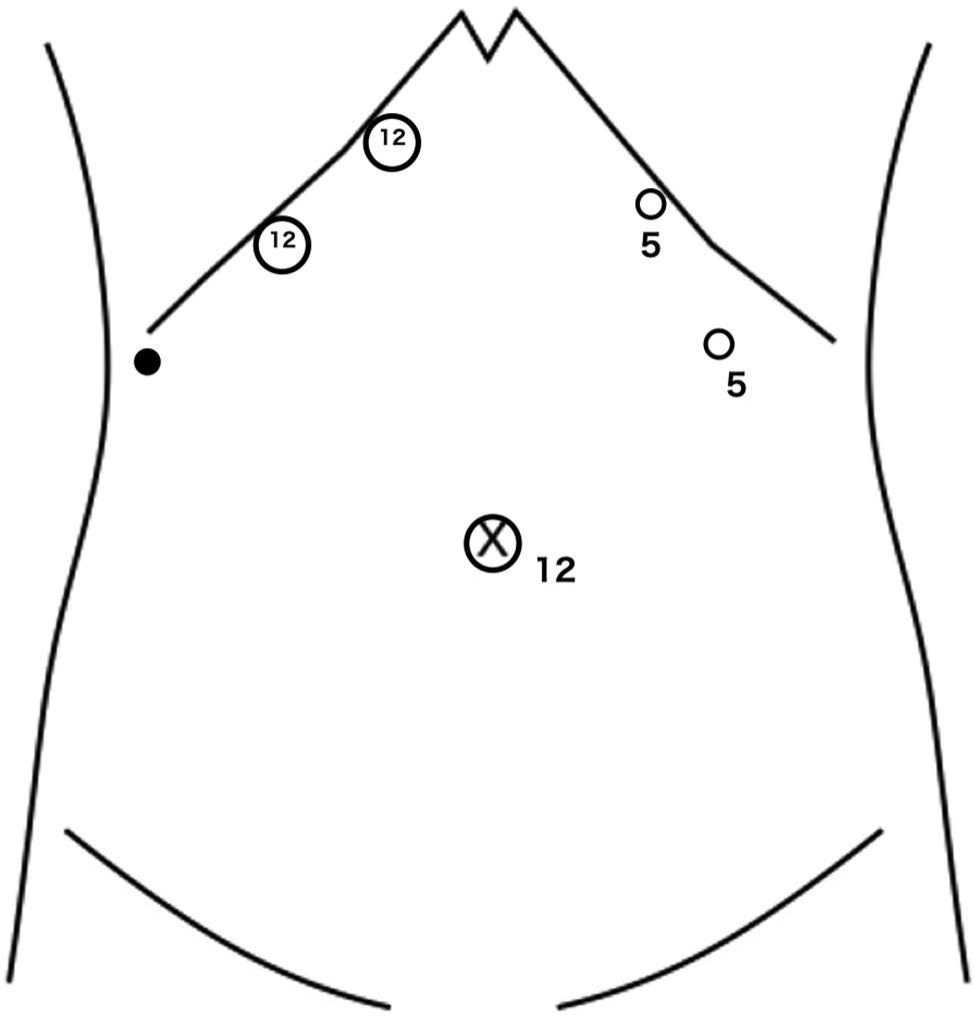
Trocar placement. The black circle indicates tourniquet for intermittent inflow occlusion. The main surgeon uses 2 12-mm trocars in the right hypochondrium for the Cavitron Ultrasonic Surgical Aspirator (CUSA) Excel system (Integra LifeSciences, Tullamore, Ireland) and a linear stapler. The specimen is extracted from the appropriately enlarged wound in the umbilicus.

**Figure 2.**
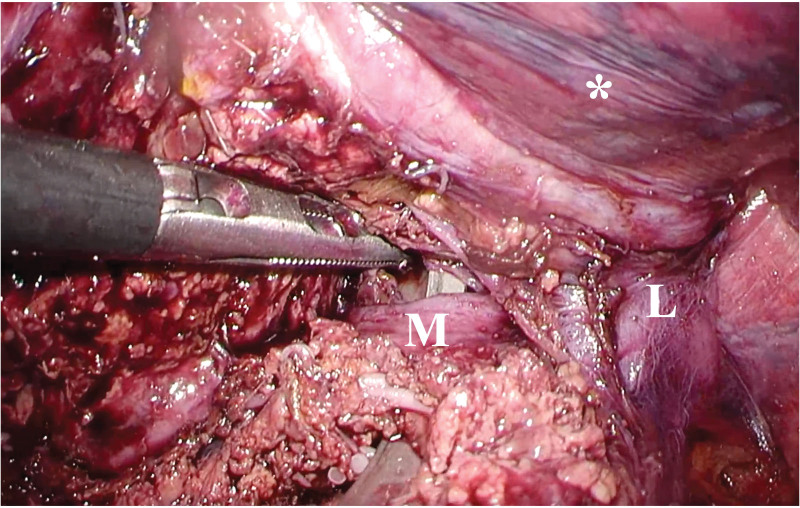
Intraoperative findings of the dorsal approach to hepatic veins. The left hepatic vein and middle hepatic vein are isolated at the root. L, left hepatic vein; M, middle hepatic vein; *, lateral segment.

In the present study, extended left hemi-hepatectomy was defined as left hemi-hepatectomy combined with middle hepatic vein resection. Additionally, whole caudate lobe was preserved in this case series. The patient was positioned supine in the reverse Trendelenburg position, but a left semi-lateral position was considered if the parenchymal transection line was distant from the midline of the body. The operation was performed with 5 trocars plus one tourniquet (Fig. 1) with fixed 10 mm Hg pneumoperitoneum pressure.^[10]^ Liver parenchyma was dissected using a Cavitron Ultrasonic Surgical Aspirator Excel system (Integra LifeSciences, Tullamore, Ireland) equipped with soft coagulation electrocautery (VIO^®^, ERBE Elektromedizin, Tuebingen, Germany). Thunderbeat (Olympus Medical Systems Corp., Tokyo, Japan) was used as the cutting energy device. Total liver inflow occlusion was employed during parenchymal transection.^[11]^ After mobilization of the lateral sector and division of the Arantius plate, the parenchyma above the Arantius plate is removed to expose the root of the left hepatic vein and to isolate the left Glissonean pedicle.^[7]^ After removing the parenchyma on the cranial side of the left Glissonean pedicle, the left Glissonean pedicle can be isolated using the Glissonean pedicle transection method. After division of the left Glissonean pedicle, the middle hepatic vein can be exposed and both the left and middle hepatic veins can be isolated (Fig. 2). Each of these veins can be isolated separately either intra- or extra-hepatically. After division of the hepatic veins, segment 4, which is the most common tumor location, can be dissected from the anterior plane of the paracaval portion of the caudate lobe by the dorsal approach, along with the hepatic hilum. Following dissection of the dorsal side of the tumor, division of the parenchyma from the anterior edge of the liver enables the anterior Glissonean branches to be divided safely, as the goal of the parenchymal transection was already identified. The specimen then is resected and removed from the enlarged wound at the umbilicus. A drainage tube is inserted from the most far right trocar site, with the tip placed at the stump of the liver and Glissonean pedicle.

### 2.2. Video case 1

A 57-year-old man was treated with lenvatinib for hepatocellular carcinoma (HCC) that was unresectable due to invasion to both the Glissonean branch of the umbilical portion and the anterior and posterior Glissonean branches. His tumor marker levels decreased thereafter along with tumor shrinkage, and the tumor became resectable after detachment from the posterior and anterior Glissonean branch. The patient underwent portal vein embolization followed by laparoscopic extended left hemi-hepatectomy.

### 2.3. Video case 2

A 75-year-old man was diagnosed with HCC in segment 4, close to the Glissonean branch of the umbilical portion, and with a high des-γ-carboxy prothrombin value. He was considered a suitable candidate for laparoscopic extended left hemi-hepatectomy.

## 3. Results

Three patients underwent laparoscopic extended left hemi-hepatectomy, none of whom required open conversion. Table 1 summarizes the perioperative outcomes. A negative surgical margin was achieved in all 3 patients. None had severe postoperative complications (Clavien-Dindo classification grade ≥ 3). The patient with sigmoid colon cancer liver metastasis underwent concomitant segment 5 partial resection, and had recurrence with intrahepatic metastasis (not cut-end recurrence) and peritoneal dissemination at 22 months after the liver resection. In the remaining 2 patients with HCC, there was no evidence of recurrence at 20 months and 10 months after resection.

**Table 1:** Perioperative outcomes.

Age (y)/sex	Diagnosis	Surgical duration, min	Blood loss, g	Complications†	Discharge, POD	Recurrence
84/female	Liver metastasis††	331*	200	None	27§	Liver, peritoneum (22 POM)
57/male (Video case 1)	HCC	277	100	None	9	None (20 POM)
75/male (Video case 2)	HCC	315	100	None	6	None (10POM)

HCC = hepatocellular carcinoma, POD = postoperative day, POM = postoperative mo.

† Clavien–Dindo classification grade ≥ 3.

†† Multiple liver metastases from sigmoid colon cancer.

* Concomitant partial resection of segment 5.

§ Long postoperative stay due to urinary tract infection.

## 4. Discussion

The dorsal approach has been reported as a useful approach in laparoscopic left hemi-hepatectomy.^[7,8]^ Because the dissection area is always located on the upper side during the dorsal approach, blood pooling is prevented at the dissection area and thus the operative field remains dry.^[8]^ Another advantage of this approach is that the middle hepatic vein can be easily exposed, as it runs close to the Arantius plate.^[8]^ In addition to these previously reported advantages of the dorsal approach, we consider that it enables early dissection of the left Glissonean pedicle and left hepatic vein. If the left Glissonean pedicle and left hepatic vein are divided first, the dorsal approach for laparoscopic extended left hemi-hepatectomy might have more advantages because it enables dissection of the dorsal margin of the tumor along with the hilar plate, and easy handling of the middle hepatic vein. The present findings demonstrated the feasibility and safety of the dorsal approach in laparoscopic extended left hemi-hepatectomy, without compromising the oncological result.

As tumors that are considered candidates for extended left hemi-hepatectomy are often located in segment 4,^[9]^ parenchymal transection along the hilar plate is required to secure the dorsal margin of the tumor. Parenchymal transection along the hilar plate from the right side of the tumor with preservation of the anterior Glissonean branch can be difficult due to the narrow parenchymal dissection plane, and may expose the tumor or cause injury of the hilar plate. In contrast, parenchymal transection from the patient left side after division of the left Glissonean pedicle and hepatic veins enables easy detection of the appropriate parenchymal cutting plane at the anterior plane of the caudate lobe.

Left hemi-hepatectomy with and without the middle hepatic vein differs in terms of the parenchymal transection line. In left hemi-hepatectomy with preservation of the middle hepatic vein, the parenchymal transection line generally extends from the anterior edge of Cantlie line to the root of the left Glissonean pedicle. In extended left hemi-hepatectomy, the dorsal goal of the parenchymal dissection is usually set closer to the anterior Glissonean branch at the hilar plate. Therefore, parenchymal dissection along the hilar plate to the anterior Glissonean branch from the patient left side is the important procedure during extended left hemi-hepatectomy. Furthermore, this procedure is often difficult in the open approach with ventral operative view. Therefore, the magnified laparoscopic caudal view has several advantages in parenchymal dissection along the hilar plate while securing the dorsal margin of the tumor. In addition, parenchymal transection from the anterior edge to secure the tumor resection margin is often difficult because the transection line is not straight, but rather curves along the hilar plate. Identification of the goal of the parenchymal transection by the dorsal approach enables simple parenchymal dissection from the anterior edge (supplemental video, http://links.lww.com/MD/L801).

The video of case 2 shows that we could preserve the root of the middle hepatic vein, and that the middle hepatic vein was divided just behind the tumor. In exposing the middle hepatic vein from the root to peripheral side just behind the tumor, we could avoid splitting injury of this vein, as described previously.^[8]^ This is a substantial advantage of the dorsal approach during left hemi-hepatectomy,^[8]^ and its usefulness has also been shown in extended left hemi-hepatectomy.

This study has some limitations. The number of patients was very small. Although none in our HCC cases experienced intrahepatic recurrence during the follow-up period, the long-term prognosis is unclear. Compression of the tumor from the dorsal side might cause trans-portal intrahepatic recurrence. Therefore, further study with a larger number of patients is required. However, the dorsal approach using magnified laparoscopic caudal operative view provides an additional operative option for laparoscopic liver resection, and may increase the safety and feasibility of laparoscopic extended left hemi-hepatectomy.

In conclusion, the dorsal approach maximizes the advantages of laparoscopic extended left hemi-hepatectomy and can be performed safely.

## Author contributions

**Conceptualization:** Katsunori Sakamoto, Kohei Ogawa

**Data curation:** Katsunori Sakamoto, Kei Tamura

**Formal analysis:** Katsunori Sakamoto, Kei Tamura

**Investigation:** Katsunori Sakamoto

**Methodology:** Katsunori Sakamoto

**Resources:** Katsunori Sakamoto

**Software:** Katsunori Sakamoto

**Supervision:** Kohei Ogawa, Kei Tamura, Masahiko Honjo, Takahiro Hikida, Chihiro Ito, Miku Iwata, Akimasa Sakamoto, Mikiya Shine, Yusuke Nishi, Mio Uraoka, Tomoyuki Nagaoka, Naotake Funamizu, Yasutsugu Takada

**Writing – original draft:** Katsunori Sakamoto

**Writing – review & editing:** Kohei Ogawa, Kei Tamura, Masahiko Honjo, Takahiro Hikida, Chihiro Ito, Miku Iwata, Akimasa Sakamoto, Mikiya Shine, Yusuke Nishi, Mio Uraoka, Tomoyuki Nagaoka, Naotake Funamizu, Yasutsugu Takada

## Supplementary Material


